# Caffeine Induces Autophagy and Apoptosis in Auditory Hair Cells *via* the SGK1/HIF-1α Pathway

**DOI:** 10.3389/fcell.2021.751012

**Published:** 2021-11-16

**Authors:** Xiaomin Tang, Yuxuan Sun, Chenyu Xu, Xiaotao Guo, Jiaqiang Sun, Chunchen Pan, Jingwu Sun

**Affiliations:** Departments of Otolaryngology-Head and Neck Surgery, The First Affiliated Hospital of University of Science and Technique of China, Hefei, China

**Keywords:** apoptosis, caffeine, autophagy, SGK1, auditory hair cells

## Abstract

Caffeine is being increasingly used in daily life, such as in drinks, cosmetics, and medicine. Caffeine is known as a mild stimulant of the central nervous system, which is also closely related to neurologic disease. However, it is unknown whether caffeine causes hearing loss, and there is great interest in determining the effect of caffeine in cochlear hair cells. First, we explored the difference in auditory brainstem response (ABR), organ of Corti, stria vascularis, and spiral ganglion neurons between the control and caffeine-treated groups of C57BL/6 mice. RNA sequencing was conducted to profile mRNA expression differences in the cochlea of control and caffeine-treated mice. A CCK-8 assay was used to evaluate the approximate concentration of caffeine. Flow cytometry, TUNEL assay, immunocytochemistry, qRT-PCR, and Western blotting were performed to detect the effects of SGK1 in HEI-OC1 cells and basilar membranes. *In vivo* research showed that 120 mg/ kg caffeine injection caused hearing loss by damaging the organ of Corti, stria vascularis, and spiral ganglion neurons. RNA-seq results suggested that SGK1 might play a vital role in ototoxicity. To confirm our observations *in vitro*, we used the HEI-OC1 cell line, a cochlear hair cell-like cell line, to investigate the role of caffeine in hearing loss. The results of flow cytometry, TUNEL assay, immunocytochemistry, qRT-PCR, and Western blotting showed that caffeine caused autophagy and apoptosis *via* SGK1 pathway. We verified the interaction between SGK1 and HIF-1α by co-IP. To confirm the role of SGK1 and HIF-1α, GSK650394 was used as an inhibitor of SGK1 and CoCl_2_ was used as an inducer of HIF-1α. Western blot analysis suggested that GSK650394 and CoCl_2_ relieved the caffeine-induced apoptosis and autophagy. Together, these results indicated that caffeine induces autophagy and apoptosis in auditory hair cells *via* the SGK1/HIF-1α pathway, suggesting that caffeine may cause hearing loss. Additionally, our findings provided new insights into ototoxic drugs, demonstrating that SGK1 and its downstream pathways may be potential therapeutic targets for hearing research at the molecular level.

## Introduction

According to the WHO’s report on hearing, more than 1.5 billion people now suffer from hearing loss worldwide, and nearly 2.5 billion people will be living with different degrees of hearing loss by 2050. Societal changes have made hearing loss more common owing to excessive exposure to loud noise and ototoxic drugs being more common (Brown et al., 2018). Hearing loss is closely associated with decreased quality of life, especially by impacting speech and language development in children and causing social problems for adults (Lasak et al., 2014). The clinical treatment of hearing loss depends on the cause and type of hearing loss. Medical therapy, surgery, amplification, or hearing implants have been used to improve the threshold (Lee and Bance, 2019). However, the effect of these clinical treatments is limited. There are still a substantial number of people suffering from cureless hearing loss. Thus, it is urgent to identify an effective method to prevent or improve hearing loss.

For all the reasons leading to hearing loss, ototoxic drugs are regarded as the major preventable factors (Liu W. et al., 2019; Gao S. et al., 2019; Guo et al., 2019; Zhang et al., 2019; Zhong et al., 2020; Fu et al., 2021b). Some general therapeutic drugs, such as antineoplastic drugs and aminoglycosides, directly kill inner ear cells, ultimately leading to serious hearing loss (He et al., 2015; Liu et al., 2016; Zhang et al., 2017; Li et al., 2018). Different drugs may damage different parts of the cochlea. Previous studies have confirmed that cisplatin damages hair cells, spiral neurons, supporting cells, and vascular veins (Gao D. et al., 2019). Additionally, aminoglycoside antibiotics can cause vestibulotoxicity, characterized by vertigo and dizziness, and cochleotoxicity. Thus, preventative treatment and mechanisms of ototoxic drugs have become one of the hot topics in hearing research.

Caffeine, as one of the most widely used drugs worldwide, is well known as a major component of common drinks, and it has an incitant effect on the nervous system, stimulating the cerebral cortex and relieving fatigue (Carolyn Brice, 2001; Mielgo-Ayuso et al., 2019). Paul et al. (2019) had found that caffeine intake protects against Parkinson’s disease. Moreover, some studies have shown that long-term coffee consumption is linked to a lower risk of type 2 diabetes (Salazar-Martinez et al., 2004; van Dam et al., 2020). In contrast, a large dose of caffeine causes a negative impact on the human body. Excessive caffeine intake can cause obvious arrhythmias, palpitations, and other cardiovascular diseases (Hartley et al., 2004; Zulli et al., 2016). Caffeine has also been demonstrated to stimulate hypersecretion of stomach glands and increase in stomach acid, leading to the formation of gastric ulcers (Kwiecien and Konturek, 2003; Liszt et al., 2017). A previous study has indicated that caffeine may have a detrimental effect on hearing recovery after a single event of acoustic trauma (Mujica-Mota et al., 2014). In contrast, some researchers have found that coffee consumption is associated with a lower risk of disabling hearing impairment in men (Machado-Fragua et al., 2021). Hearing loss is one of the major symptoms in Ménière’s disease. Restriction of salt, caffeine, and alcohol intake is recommended to patients with Ménière’s disease as a first-line treatment (Hussain et al., 2018). Caffeine may result in a reduction in the blood supply to the inner ear, which may worsen the symptoms of patients. Overall, it is not clear whether caffeine has a direct effect on hearing cells; thus, exploring the regulatory mechanism of caffeine may provide new insights into the protection of hearing loss.

Autophagy, a dynamic mechanism of cellular defense and self-protection, is an effective mechanism that promotes cell survival by removing impaired proteins and nonfunctional organelles (Mizushima et al., 2001). Autophagy have been reported in many previous studies to protect the cochlear hair cells (He et al., 2017; He et al., 2020; Zhou et al., 2020; He et al., 2021) and spiral ganglion neurons (Liu et al., 2021). Apoptosis is the most well-known form of programmed cell death in the inner ear cochlea (Sun et al., 2014; Yan et al., 2018; He et al., 2019; Ding et al., 2020; Fu et al., 2021a; Cheng et al., 2021) and is responsible for removing aging, damaged, or mutated cells. Apoptosis and autophagy are functionally interrelated. Caffeine has been revealed to induce apoptosis and mitochondrial dysfunction in the neonatal rat brain (Kasala et al., 2020). Moreover, caffeine is regarded as a potent stimulator of hepatic autophagic flux in mice (Sinha et al., 2014). Endoplasmic reticulum stress has been found to mediate autophagy, which enhances caffeine-induced apoptosis in hepatic stellate cells (Li et al., 2017). However, it remains unknown whether caffeine regulates autophagy and apoptosis in hair cells.

In the present study, we explored the effect of caffeine on HEI-OC1 cells and cochlear hair cells as well as the underlying mechanism to better understand caffeine ototoxicity in hearing.

## Materials and methods

### Animals

Twenty-seven C57BL/6 mice (weight 20 g each, 2 months, male) were purchased from the Model Animal Research Center of Nanjing University, China. The animal research was completed with the approval of the Ethics Board of the first affiliated hospital of USTC (2021-N(A)-019). These mice were kept in a specific pathogen-free environment, where humidity was maintained in the range of 50%–60% and temperature was at 25°C. These mice were randomly decided into four groups: group I received daily normal saline injection of 0.2 ml as control (*n* = 8); group II received daily caffeine (Sigma-Aldrich, St. Louis, MO, USA) injection of 120 mg/ kg for 7 days (*n* = 8); group III received daily caffeine injection of 120 mg/kg for 14 days (*n* = 8); group IV received daily caffeine injection of 20 mg/ kg for 14 days (*n* = 3). These were sacrificed by overdose of ketamine after Auditory Brainstem Response (ABR) tests.

Twenty-four Sprague-Dawley (SD) rats [postnatal day 3 (P3)] were purchased from the Model Animal Research Center of Nanjing University, China. These SD rats were sacrificed by overdose of ketamine. Cochlear basilar membranes were cultured in Neurobasal medium (Gibco, Grand Island, NY, USA) after being gently isolated from SD rats. The 24 SD rats were equally allocated to two experiments. In one, 12 neonatal SD rats were divided into four groups: no caffeine treated as control group (*n* = 3); 1 mM caffeine group (*n* = 3); 5 mM caffeine group (*n* = 3); and 10 mM caffeine group (*n* = 3). In the other, 12 neonatal SD rats were divided into four groups, as shown in Figure 6C: no caffeine treated as control group (*n* = 3); 10 μM GSK650394 group (*n* = 3); 10 mM caffeine group (*n* = 3); and 10 μM GSK650394 + 10 mM caffeine group (*n* = 3).

### Cell culture and drug treatment

The House Ear Institute-Organ of Corti 1 (HEI-OC1) cell line has been extensively used in many previous reports (Guan et al., 2016; He et al., 2016; Yu et al., 2017) and was a gift from professor Hao Xiong in the Sun Yat-sen University (Xiong et al., 2019). HEI-OC1 cells were cultured under permissive conditions (33°C, 10% CO_2_) in DMEM (Servicebio, Wuhan, China) supplemented with 10% fetal bovine serum (Gibco, Sydney, Australia). Caffeine was added to the culture medium at a concentration of 1, 5, 10, and 20 mM. In order to confirm the role of SGK1 (serum and glucocorticoid-induced protein kinase 1) in HEI-OC1 cells after caffeine treatment, GSK650394, a SGK1 inhibitor, was used (Peng et al., 2012): no caffeine treated as control group; 10 μM GSK650394 group; 10 mM caffeine group; and 10 μM GSK650394 + 10 mM caffeine group. In order to confirm the role of HIF-1α (hypoxia inducible factor-1) in HEI-OC1 cells, we divided HEI-OC1 cells into four groups (Figure 5F): no caffeine treated as control group; 100 μM CoCl_2_ group; 10 mM caffeine group; and 100 μM CoCl_2_ + 10 mM caffeine group (Zhao et al., 2020). In order to verify whether autophagy leads to apoptosis, we divided HEI-OC1 cells into four groups (Supplementary Figure S3): no caffeine treated as control group; 5 mM 3-MA group; 10 mM caffeine group; and 5 mM 3-MA + 10 mM caffeine group.

### Auditory brainstem response

Tests were performed under general anesthesia induced through injection of 100 mg/kg ketamine. ABR tests were conducted using a Tucker-Davis Technology System hardware and software (Alachua, NY, USA). C57BL/6 mice were subcutaneously inserted with subdermal needle electrodes at the vertex, below the left ear (reference), and on the right ear (ground) after being anesthetized. ABR tests were measured at frequencies of 8, 16, and 32 kHz. In order to obtain the average response to 1,024 stimuli, the stimulus sound was decreased from 90 to 10 dB SPL, whose intensity was reduced by 10 dB at intervals near the threshold. As soon as the electrophysiological response to the stimulus sound disappeared, we measured the minimum stimulus sound that evoked a response as the hearing threshold of the mouse tested at this frequency.

### Immunofluorescence

Basilar membranes and cells were isolated and fixed in 4% paraformaldehyde. Basilar membranes were divided into three segments (apex, middle, and base) and mounted on glass slides. Then, they were incubated in 0.5% Triton X-100 for 20 min at room temperature. After incubation in 10% goat serum for blocking nonspecific antibody binding for 30 min at room temperature, the samples were incubated with the primary antibody (information in Table1) overnight at 4°C. After washing three times (5 min each), the tissues were incubated with the secondary antibody (Jackson, West Grove, PA, USA, 1:100) at room temperature for 1 h. Basilar membranes needed to be incubated with phalloidin (Yeasen, Shanghai, China, 1:100) containing fluorescein isothiocyanate at room temperature for another 1 h. The nuclei were stained by DAPI (Biosharp, Shanghai, China) for 5 min. Images were captured by using Zeiss LSM800.

**TABLE 1:** Primers in this study.

Gene	Forward	Reserve
GAPDH	GGC​ATT​GTG​GAA​GGG​CTC​AT	TGT​CAT​CAT​ACT​TGG​CAG​GTT​TC
SGK1	GCC​AAG​TCC​CTC​TCA​ACA​AAT​CA	GTG​CCT​AGC​CAG​AAG​AAG​AAC​CTT​T
p62	CCT​CAG​CCC​TCT​AGG​CAT​TG	TTC​TGG​GGT​AGT​GGG​TGT​CA
Caspase3	GAG​CTT​GGA​ACG​GTA​CGC​TA	GCG​AGA​TGA​CAT​TCC​AGT​GC
LC3B	AGA​GCG​ATA​CAA​GGG​GGA​GA	TGC​AAG​CGC​CGT​CTG​ATT​A
Bcl-2	CAG​CCT​GAG​AGC​AAC​CCA​AT	TAT​AGT​TCC​ACA​AAG​GCA​TCC​CAG
Bax	TGG​AGC​TGC​AGA​GGA​TGA​TT	TCT​TGG​ATC​CAG​ACA​AGC​AGC

### Immunohistochemistry

The sections were dewaxed and deparaffinized in xylene and rehydrated in graded alcohol solutions. Then, the sections were heated for 30 min in Tris–EDTA buffer by microwave oven. Subsequently, the slides were stained with hematoxylin and eosin (HE) or primary antibodies for SGK1 (information in Table 1) and their respective secondary antibodies. Before dehydration and mounting, the sections were counter-stained with hematoxylin. Images were performed with an Olympus microscope camera (Tokyo, Japan). The stria vascularis thickness of cochlea was measured by Olympus cellSens Standard software (Olympus life science, Tokyo, Japan).

### RNA-Seq

Total RNA was extracted using TRIzol reagent kit (Invitrogen, Carlsbad, CA, USA) according to the manufacturer’s protocol. RNA quality was assessed on an Agilent 2100 Bioanalyzer (Agilent Technologies, Palo Alto, CA, USA) and checked using RNase free agarose gel electrophoresis. After total RNA was extracted, mRNA was enriched by Oligo (dT) beads, while prokaryotic mRNA was enriched by removing rRNA by Ribo-Zero™ Magnetic Kit (Epicentre, Madison, WI, USA). Then the enriched mRNA was fragmented into short fragments using fragmentation buffer and reverse transcripted into cDNA with random primers. Second-strand cDNA were synthesized by DNA polymerase I, RNase H, dNTP, and buffer. Then the cDNA fragments were purified with the QIAquick PCR extraction kit (Qiagen, Venlo, Netherlands), end repaired, poly(A) added, and ligated to Illumina sequencing adapters. The ligation products were size selected by agarose gel electrophoresis, PCR amplified, and sequenced using Illumina HiSeq 2500 by Gene Denovo Biotechnology Co. (Guangzhou, China).

### CCK-8

Cell viability was detected using CCK-8 kits (Topscience, Shanghai, China) according to the manufacturer’s protocols. Briefly, 5,000 cells were plated into a 96-well flat-bottom plate and incubated overnight under permissive conditions. After drug treatment in 100 µl culture medium, 10 µl CCK-8 was added for 1 h at 37°C. The optical density (OD) values were measured at 450 nm by an ELISA reader (Thermo Multiskan Mk3, Waltham, MA, USA). The blank underwent the same procedure, but without cell seeding, whereas the negative control was just treated without drugs. The relative viability was calculated as (OD experiment–OD blank)/(OD control–OD blank) × 100%.

### Flow cytometry

HEI-OC1 cells (5 × 10^3^ cells/well) were seeded into six-well plates overnight. Cells were collected after being digested with trypsin. An Annexin FITC/PI Apoptosis Detection Kit (Yeasen, Shanghai, China) was used to examine the rate of apoptosis. Data analysis was performed by using NovoCyte (Agilent, Santa Clara, CA, USA).

### TUNEL assay

The experiment was conducted with the One Step TUNEL Apoptosis Assay Kit (Beyotime, Shanghai, China) according to the manufacturer’s instructions. After being fixed in 4% paraformaldehyde for 20 min, HEI-OC1 cells were then incubated in 0.5% Triton X-100 for 5 min. Next, samples were labeled with 50 μl TUNEL reaction mixture and incubated at room temperature for 1 h in the dark. After washing, slides were immediately examined under a Leica microscope (DMi8). The percentage of apoptotic cells was calculated as (TUNEL-positive cells/total cells) × 100%. All assays were performed in triplicate.

### Electron microscopy

HEI-OC1 cells were fixed in TEM fixative (Servicebio, Wuhan, China) for 24 h and then fixed at 4°C for preservation and transportation. The 1% agarose solution was prepared by heating and dissolving in advance. Before agarose solidification, the precipitation was suspended with forceps and wrapped in the agarose. Agarose blocks with samples avoid light post fixed with 1% OsO_4_ (Ted Pella Inc., CA, USA) for 2 h at room temperature. An ethanol dehydration process (series of 30%, 50%, 70%, 80%, 95% and two changes of 100% ethanol) followed by a 20-min immersion in acetone was performed before the final EPON resin. The resin blocks were cut to 60–80 nm thin on an ultramicrotome (Leica, Germany), and the tissues were fished out onto 150 mesh cuprum grids with formvar film. Two percent of uranium acetate was saturated with alcohol solution to avoid light staining for 8 min, then 2.6% lead citrate to avoid CO_2_ staining for 8 min. The cuprum grids are observed under TEM (Hitachi, HT7800), and images were taken.

### Quantitative real-time polymerase chain reaction analysis

Total RNA was isolated from cells by using TRIzol reagent (Takara Bio, Tokyo, Japan). On the basis of the instructions of the reverse transcriptase kit (Takara, Tokyo, Japan), cDNA was synthesized using 2 μg of the total RNA in TProfessional Thermocycler (Biometra, Berlin, Germany). Then, cDNA samples were subjected to qRT-PCR for 40 cycles by using TB Green™ Premix Ex Taq™ II (Takara, Tokyo, Japan) in Roche LightCycler 96 (Roche, Basel, Switzerland). Primers (Table 1) were designed with the approval of the Sango Biotech Co. Ltd. (Shanghai, China). GAPDH was used as an internal control. The results were calculated using the comparative cycle threshold (ΔΔCt) method.

### Western blot analysis

First, total protein was extracted from HEI-OC1 cells and basilar membrane by using RIPA buffer (Beyotime, Shanghai, China). Almost 10 μg of crude protein was denatured and electrophoresed on 12.5% SDS-PAGE gels. Proteins were transferred onto PVDF membranes by electro-blotting after electrophoretic separation, followed by blocking for 15 min at room temperature in Protein Free Rapid Blocking Buffer (Epizyme, Shanghai, China). The blots were incubated with SGK1, HIF-1α, P62, Bcl-2, Bax, Cleaved-Caspase3, and LC3B (information in Table 2) primary antibodies at 4°C overnight. After washing with PBS-T, membranes were hybridized with an appropriate secondary antibody (Abmart, Shanghai, China) at room temperature for 1 h. Lastly, images of the Western blot bands were performed with chemi Capture (Clinx, Shanghai, China) and the intensity in each group was measured with ImageJ. GAPDH was used as an internal control.

**TABLE 2:** Antibodies in this study.

	Ratio	Brand	Art.NO
WB antibody			
GAPDH	1:1000	CST	5174
HIF-1α	1:1000	CST	36169
SGK1	1:500	Beyotime	AF1909
p62	1:1000	Boster	PB0458
Cleaved-Caspase3	1:1000	CST	9664
LC3B	1:1000	Proteintech	18725
Bcl-2	1:1000	Abcam	ab194583
Bax	1:1000	Abcam	ab182734
IF antibody			
SGK1	1:100	Abcam	ab43606
TUBB3	1:100	Proteintech	CL488-66240
p62	1:100	Boster	PB0458
IHC antibody			
SGK1	1:100	Beyotime	AF1909

### Statistical analysis

Data are shown as the mean ± SD, and all experiments were repeated at least three times. Statistical analysis was conducted using GraphPad Prism 6. Two-tailed, unpaired Student’s t-tests were used to determine statistical significance when comparing two groups, and one-way ANOVA followed by a Dunnett multiple-comparison test was used when comparing more than two groups. A value of *p* < 0.05 was considered statistically significant.

## Results

### Caffeine destroys cochlear hair cells, stria vascularis, and spiral ganglion neurons in C57BL/6 mice

**FIGURE 1:**
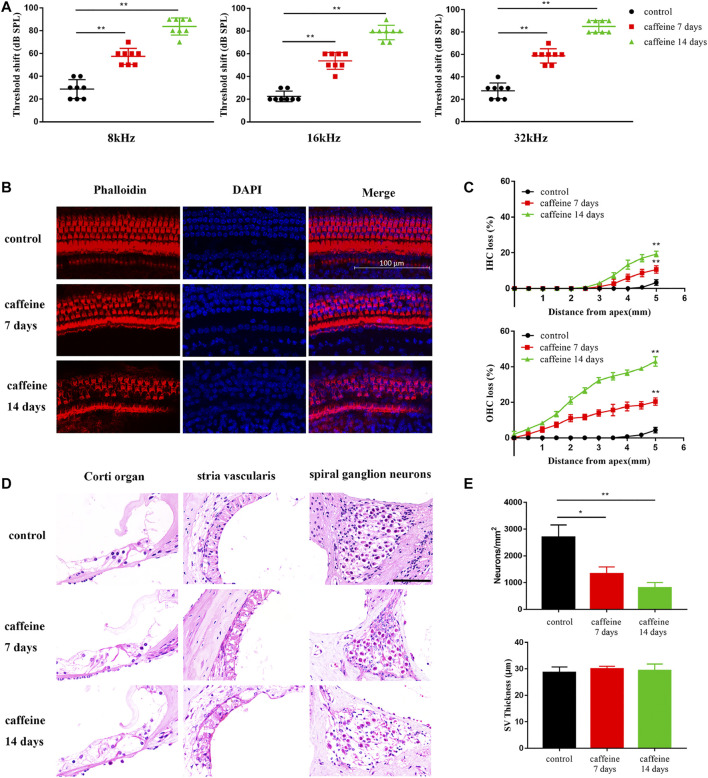
Caffeine destroys cochlear hair cells, stria vascularis, and spiral ganglion neurons in C57BL/6 mice. **(A)** ABR threshold shifts measured in C57BL/6 mice treated with 120 mg/kg caffeine for 7 or 14 days. The control mice were treated with the same volume of normal saline. Data are presented as individual points and means ± SD, **p < 0.01. **(B)** Images of middle membrane stained with phalloidin (red). DAPI (blue) was used to stain the nuclei. Scale bar: 100 μm. **(C)** Hair cell counts in C57BL/6 mice treated with 120 mg/kg caffeine at 7 and 14 days and control mice treated with normal saline. N = 3 in each group. Data are presented as individual points and means ± SD, **p < 0.01. **(D)** Images of Corti organ, spiral ganglion neuron, and stria vascularis stained with HE. Scale bar: 100 μm. **(E)** Spiral ganglion neuron counts at the middle cochlear turn in each group. N = 3 in each group. Data are presented as individual points and means ± SD, **p < 0.01, *p < 0.05.

Two-month-old C57BL/6 mice were intraperitoneally injected with caffeine 120 mg/kg/day caffeine for 7 and 14 days, while mice in the control group were treated with normal saline. After 7 and 14 days of injection, auditory threshold shifts in the caffeine group significantly increased at 8, 16, and 32 kHz (Figure 1A). However, compared to the control group, auditory threshold shifts were not significantly difference after 20 mg/kg caffeine injection (Supplementary Figure S1). These results suggested that the effect of caffeine on hearing might be dose-dependent. To explore the position of caffeine damage, basilar membranes were stained with TRITC-phalloidin (Figure 1B), and paraffin sections of cochlea were stained with HE (Figure 1D). According to these results, caffeine caused disorder of the Corti organ and the loss of spiral ganglion neurons and hair cells (Figures 1C,E). Moreover, we also found that caffeine did not lead to significant stria vascularis damage.

### Caffeine induces autophagy and apoptosis and increases the expression of SGK1 in the cochlea of C57BL/6 mice

**FIGURE 2:**
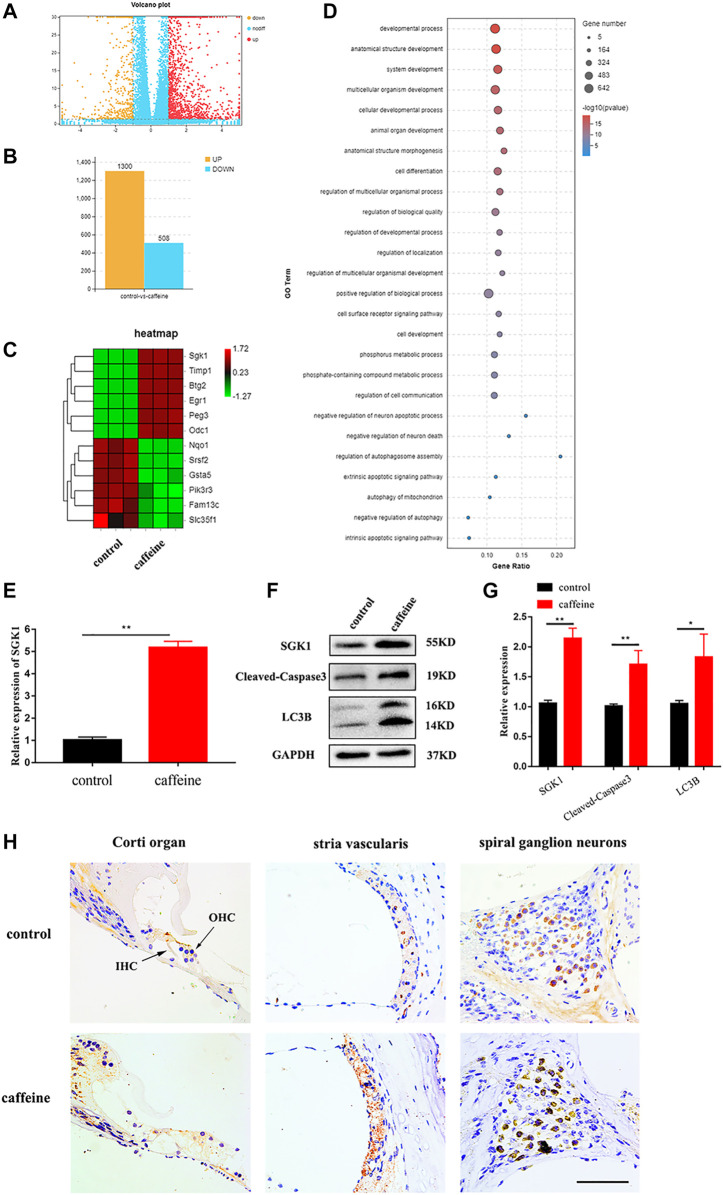
Differentially expressed mRNA in the cochlea of C57BL/6 mice with 120 mg/kg caffeine and control. **(A)** Volcano plot showing the differentially expressed mRNAs in caffeine (120 mg/kg/day, continuous injection for 14 days) treatment and control group (normal saline, continuous injection for 14 days). **(B)** The number of differentially expressed mRNAs. **(C)** Heat map showing hierarchical clustering of differentially expressed mRNAs. Red indicates high relative expression, and green represents low relative expression. **(D)** Functional enrichment of differentially expressed mRNAs. **(E)** Different mRNA levels of SGK1 in the caffeine and control groups were confirmed by qRT-PCR. Data are shown as means ± SD, **p < 0.01. **(F–G)** Western blot analysis the expression of SGK1, Cleaved-Caspase3, LC3B, and GAPDH in cochlea. Data are shown as means ± SD, **p < 0.01, *p < 0.05. **(H)** Immunohistochemistry (IHC) for SGK1 in the organ of Corti, spiral ganglion neuron, and stria vascularis of C57BL/6 mice. Scale bar, 100 μm.

To identify the mechanism of caffeine on hearing loss, cochlear tissues from the control and caffeine-treated C57BL/6 mice were collected for RNA sequencing, and a volcano plot (Figure 2A) was constructed to show all the molecules detected. Transcriptome analysis showed 1,300 upregulated and 508 downregulated mRNAs in caffeine-treated mice compared to the control group (Figure 2B). Heat maps (Figure 2C) were generated using the differentially expressed mRNAs. Bubble showed enrichments of various functional categories (Figure 2D). The expression change of SGK1, the most differentially expressed gene, was further confirmed by qRT-PCR (Figure 2E), which was consistent with the RNA-seq analysis. Additionally, the protein expression of SGK1, Cleaved-Caspase3, and LC3B was evaluated by Western blot analysis. The results showed that the expression of SGK1, Cleaved-Caspase3, and LC3B II/I markedly increased in the cochlea after caffeine injection (Figures 2F,G). To evaluate the major expression location of SGK1, we investigated the expression levels of SGK1 in C57BL/6 mouse cochlea through immunohistochemical staining. SGK1 was mainly expressed in the Corti organ, stria vascularis, and spiral ganglia (Figure 2H), which indicated that the expression of SGK1 increased in the cochlea after caffeine injection, suggesting that caffeine may induce autophagy and apoptosis in the cochlea.

### Caffeine induces apoptosis and autophagy in HEI-OC1 cells

**FIGURE 3:**
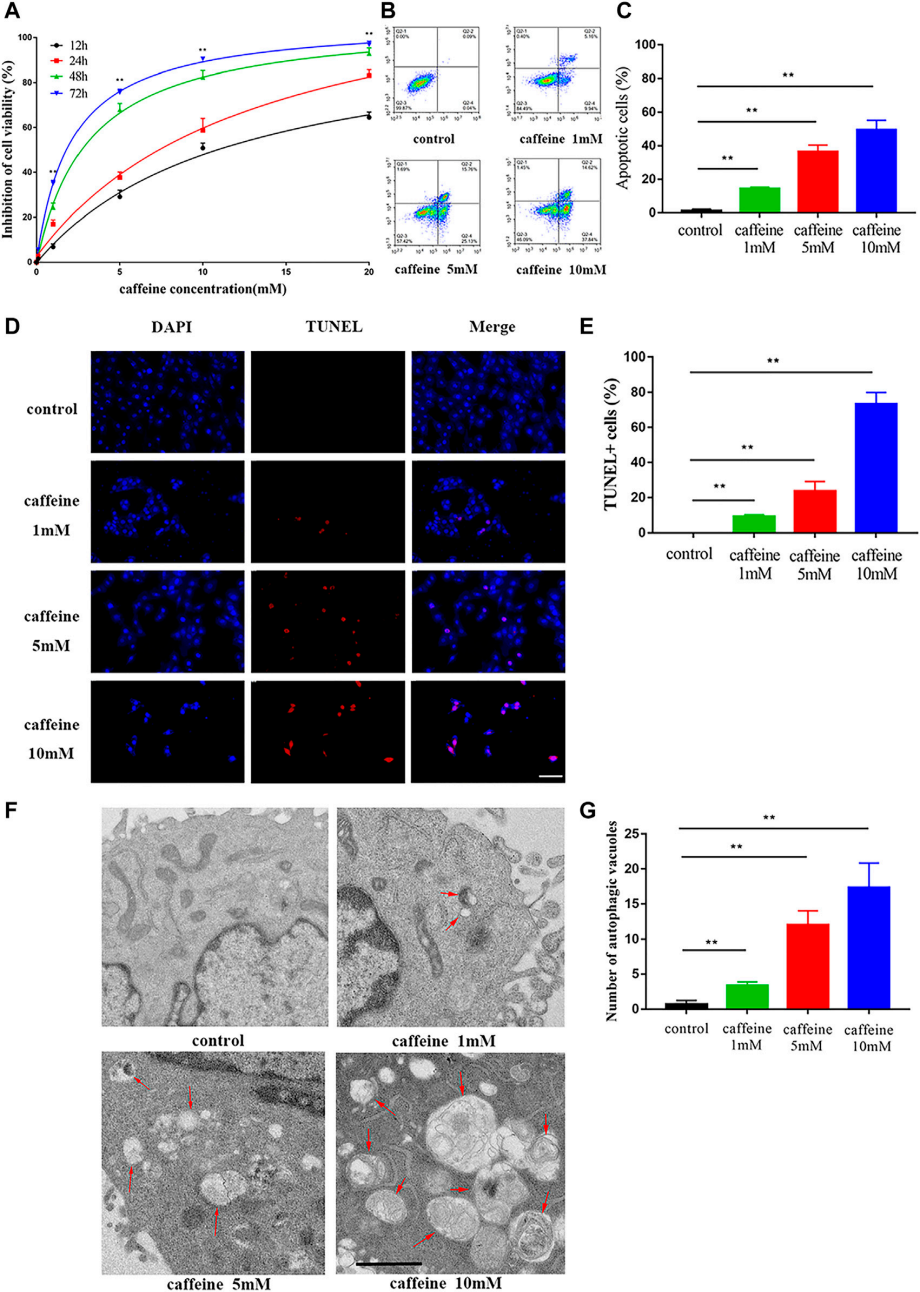
Caffeine induced autophagy and apoptosis in HEI-OC1. **(A)** CCK-8 kit was used to measure cell viability in HEI-OC1 cells after different incubation times with varying concentrations of caffeine (from 0 to 20 mM). **(B–C)** The percent of apoptosis after 24 h of caffeine treatment with different concentrations was measured by flow cytometry. Data are shown as means ± SD, **p < 0.01. **(D–E)** TUNEL (red) and DAPI (blue) double staining showing the apoptotic HEI-OC-1 cells after different treatments. Scale bar: 100 μm. Data are shown as means ± SD, **p < 0.01. **(F–G)** Electron microscope analysis for evaluating autophagy in HEI-OC1 cells. Scale bar: 1 μm. Data are shown as means ± SD, **p < 0.01.

HEI-OC1 cells were used to investigate the effect of caffeine. Cells were treated with different caffeine concentrations (0, 0.1, 1, 5, 10, and 20 mM) for different times (12, 24, 48, and 72 h), and the results showed that caffeine inhibited HEI-OC1 cell viability in a time- and dose-dependent manner. When the concentration was greater than 1 mM, the inhibitory effect of caffeine on HEI-OC1 cells was markedly increased (Figure 3A). Moreover, the cell viability inhibition of HEI-OC1 at 10 mM was similar to that of 20 mM when treated for 72 h. Based on these results, treatments with 0, 1, 5, and 10 mM caffeine for 24 h were selected as the conditions in subsequent experiments. Dead cells were labeled by PI, and the cells undergoing apoptosis were labeled by Annexin V (Figures 3B,C). The rate of apoptosis in HEI-OC1 cells treated with 1 mM (14.25 ± 0.55%), 5 mM (36.38 ± 2.30%), and 10 mM (49.40 ± 3.34%) caffeine was significantly higher than that in the control group (1.20 ± 0.57%). HEI-OC1 cells in each group were examined by TUNEL assays in order to confirm the apoptosis effect. The percentage of TUNEL-positive cells was positively correlated with the concentration of caffeine (Figures 3D,E). Transmission electron microscope imaging showed that the caffeine-treated cells had more autophagic vacuoles (double membrane-bound autophagosomes) than the control cells, indicating that autophagy levels increase after caffeine treatment (Figures 3F,G). Taken together, these results suggested that caffeine induces apoptosis and autophagy in HEI-OC1 cells.

### Caffeine induces apoptosis and autophagy *via* SGK1/HIF-1α pathway in HEI-OC1 cells

**FIGURE 4:**
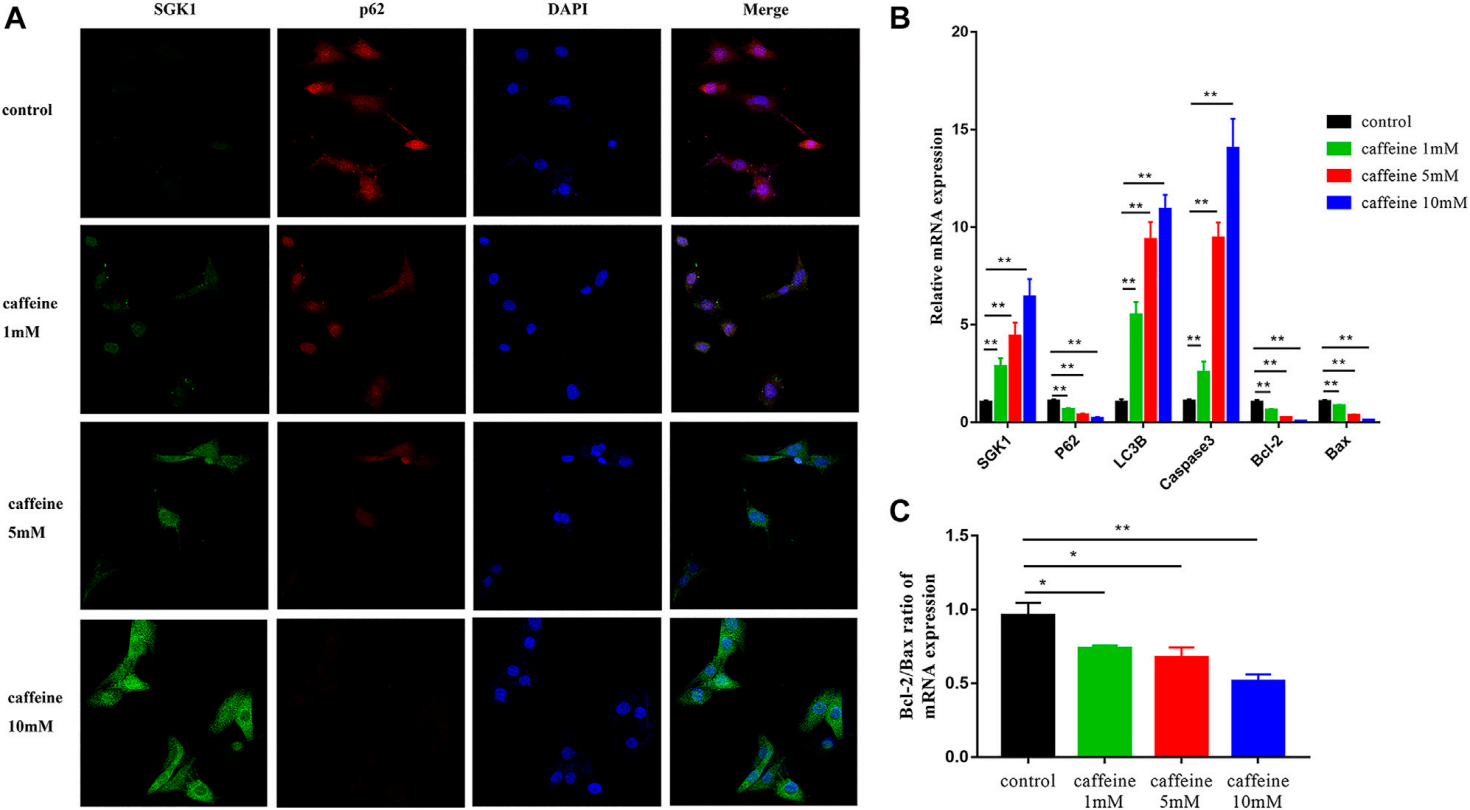
Caffeine affected the expression of SGK1, autophagy, and apoptosis-related genes. **(A)** Immunofluorescence of p62 (red) and SGK1 (green) in HEI-OC1 cells after 24 h of caffeine treatment with different concentrations (0, 1, 5, 10 mM). DAPI (blue) was used to stain the nuclei. Scale bar: 100 μm. **(B)** qRT-PCR was used to analyze the mRNA expression of SGK1, p62, LC3B, and Caspase3 in HEI-OC1 cell lines after 24 h caffeine treatment with different concentrations (0, 1, 5, 10 mM), and GAPDH was used as the internal control. Experiments were repeated three times, and data are shown as the means ± SD, **p < 0.01. **(C)** Bcl-2/Bax ratio of mRNA expression in HEI-OC1 cells. Data are shown as means ± SD, **p < 0.01.

**FIGURE 5:**
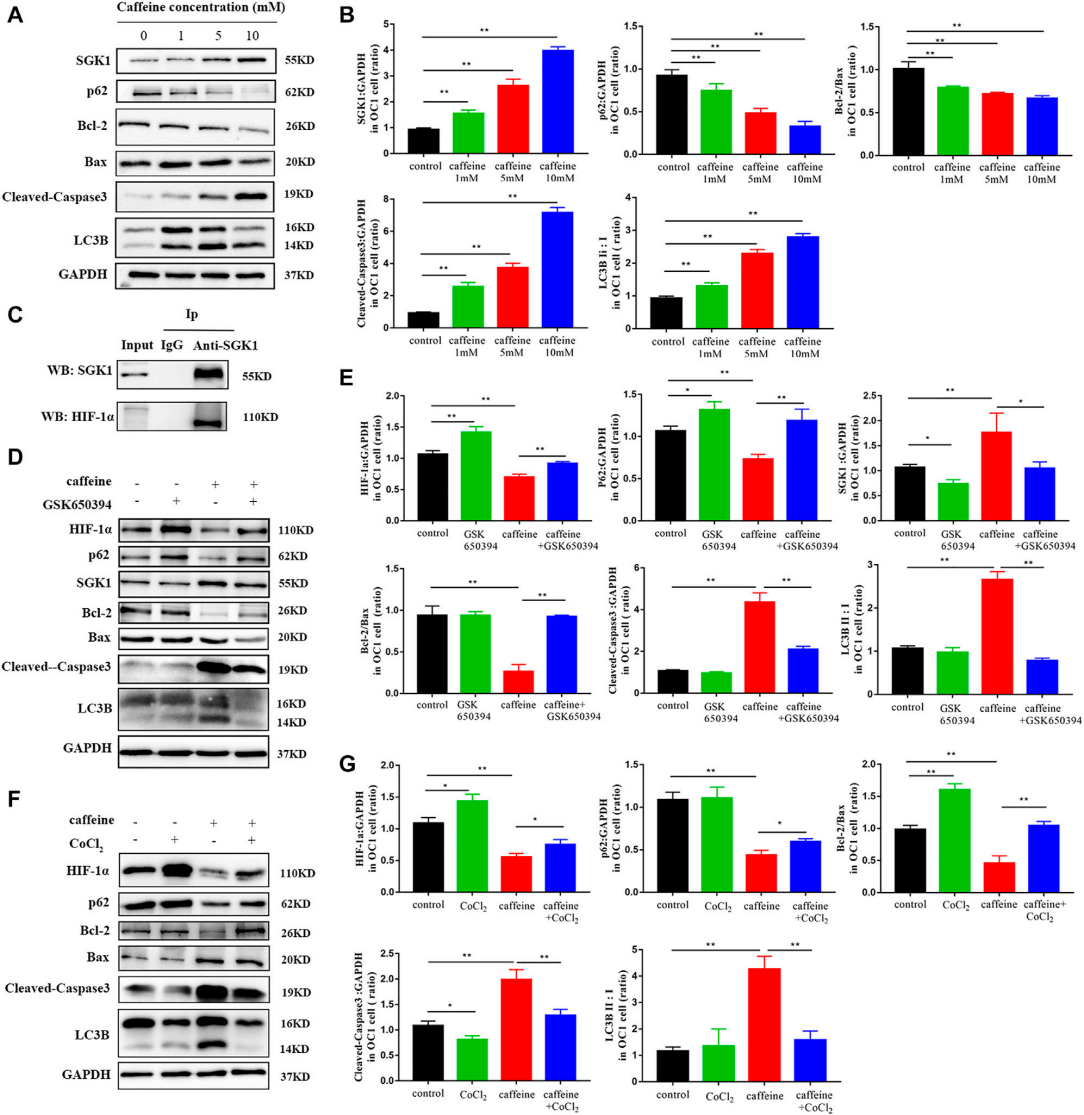
Caffeine induced autophagy and apoptosis *via* the SGK1/HIF-1α signaling pathway. **(A)** Western blotting showed changes of SGK1, p62, LC3B, Bcl-2, Bax, and Cleaved-Caspase3 in HEI-OC1 cell lines after a 24-h caffeine treatment with different concentrations, and GAPDH was used as the internal control. **(B)** Quantification of the Western blot in SGK1, p62, LC3B, Bcl-2, Bax, and Cleaved-Caspase3. Experiments were repeated three times, and data are shown as the mean ± SD, **p < 0.01. **(C)** Co-immunoprecipitation (Co-IP) analysis of SGK1 and HIF-1α protein interaction. **(D)** Western blot assay was employed to investigate the expressions of HIF-1α, SGK1, P62, LC3B, and Cleaved-Caspase3 in HEI-OC1 cells after 24 h treatment with or without caffeine and GSK650394. GAPDH was used as the internal control. **(E)** Quantification of the Western blot in HIF-1α, SGK1, P62, LC3B, Bcl-2, Bax, and Cleaved-Caspase3. Experiments were repeated three times. Data are shown as means ± SD, **p < 0.01, *p < 0.05. **(F)** Western blot assay was employed to investigate the expression of HIF-1α, P62, LC3B, and Cleaved-Caspase3 in HEI-OC1 cells after a 24-h treatment with or without caffeine and CoCl_2_. GAPDH was used as the internal control. **(G)** Quantification of the Western blot in **(F)**. Experiments were repeated three times. Data are shown as means ± SD, **p < 0.01, *p < 0.05.

We next investigated the role of SGK1 in HEI-OC1 cells by detecting the expression of SGK1 and p62 in HEI-OC1 cells by immunofluorescence. As the concentration of caffeine increased, the fluorescence intensity of SGK1 increased and the fluorescence intensity of p62 decreased, which suggested that the expression of SGK1 and p62 had a consistent trend (Figure 4A). Furthermore, we analyzed the mRNA and protein levels of apoptotic and autophagic markers to explore the involved signaling pathway. After caffeine treatment, qRT-PCR (Figures 4B,C) and Western blot analyses (Figures 5A,B) showed that the expressions of SGK1, LC3B, and caspase3 significantly increased, while the expressions of p62 and the Bcl-2/Bax ratio decreased. Thus, these findings suggested that caffeine may induce apoptosis, induce autophagy, and increase the expression of SGK1. Next, we verified the interaction between SGK1 and HIF-1α by co-IP (Figure 5C). GSK650394 is a known SGK1 inhibitor. To further confirm the role of SGK1, we generated a control group, a GSK650394-treated group, a caffeine-treated group, and a GSK650394 + caffeine-treated group. Compared to caffeine treatment alone, Western blot analyses demonstrated that GSK650394 + caffeine treatment increased the expression of HIF-1α and p62 as well as the Bcl-2/Bax ratio but inhibited the expression of SGK1, Cleaved-Caspase3, and LC3B II/I (Figures 5D,E). Next, we took advantage of CoCl_2_, an inducer of HIF-1α, to explore the role of HIF-1α in HEI-OC1 cells after caffeine treatment. The Western blot data showed that CoCl_2_ increased the expression of p62 and the Bcl-2/Bax ratio after caffeine treatment, while it inhibited the expression of SGK1, Cleaved-Caspase3, and LC3B II/I (Figures 5F,G). Additionally, we also found that CoCl_2_ did not mediate the expression of SGK1 (Supplementary Figure S2), indicating that HIF-1α might be a downstream signaling molecule of SGK1. To explore the relationship between autophagy and apoptosis, 3-methyladenine (3-MA) was used as an autophagy inhibitor. After treatment with 3-MA and caffeine, the expression of Cleaved-Caspase3 decreased and the ratio of Bcl-2/Bax increased, suggesting that autophagy may lead to apoptosis when HEI-OC1 cells are treated with caffeine (Supplementary Figure S3).

### Caffeine activates SGK1 to destroy hair cells and nerve fibers in P3 SD rats

**FIGURE 6:**
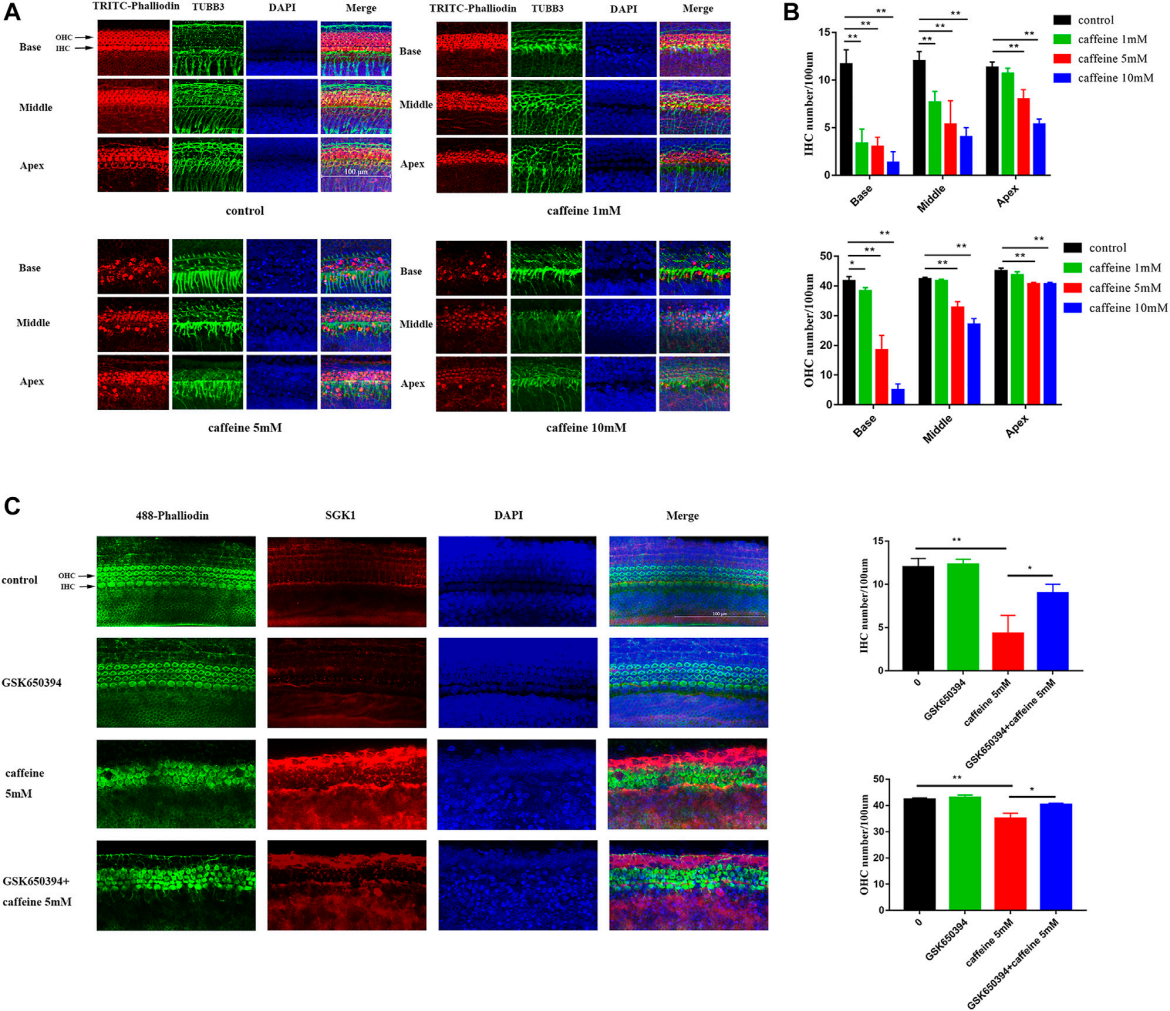
Caffeine activates SGK1 to destroy hair cells and nerve fibers in P3 SD rats. **(A–B)** The basilar membranes of SD newborn mouse were stained with fluorescent phalloidin (red) and TUBB3 (green). DAPI (blue) was used to stain the nuclei. Scale bar: 100 μm. Data are shown as means ± SD, **p < 0.01, *p < 0.05. **(C–D)** The middle turn of basilar membranes was stained with fluorescent phalloidin (green) and SGK1 (red). DAPI (blue) was used to stain the nuclei. Scale bar: 100 μm. Data are shown as means ± SD, **p < 0.01, *p < 0.05.

Cultured neonatal rat basilar membrane *in vitro* is an important hearing research model. Cochlear basilar membranes were gently isolated from P3 SD rats and used for experiments after 24 h in culture. After treatment with different concentrations of caffeine, hair cells were labeled with TRITC–phalloidin and labeled auditory nerve fibers were labeled with the 488-TUBB3 antibody. Microscopic analysis showed that caffeine caused disorder and loss of hair cells, especially inner hair cells (Figures 6A,B). However, we also found that caffeine caused disorder of auditory nerve fibers. Additionally, we confirmed the role of SGK1 in the basilar membrane of neonatal SD rats (Figure 6C). GSK650394 effectively protected hair cells from caffeine damage. These results indicated that inhibition of SGK1 might be a potential target for protecting hair cell loss, especially inner hair cells (Figure 6D).

## Discussion

Absorption of caffeine is nearly complete within 45 min after ingestion, with caffeine blood levels peaking after 15 min to 2 h (Nehlig, 2018). The half-life of caffeine in humans ranges from 2 to 12 h, mainly due to interindividual differences in absorption and metabolism (Benowitz, 1990). Caffeine metabolism is greatly reduced during pregnancy, particularly in the third trimester, when the half-life can be as long as 15 h (van Dam et al., 2020). In addition, caffeine penetrates throughout the body and crosses the blood–brain barrier. Thus, caffeine is closely related to neurologic disease. Previous studies have found that caffeine might reduce the risk of Parkinson’s disease and contribute to insomnia (Lara, 2010; Qi and Li, 2014). Hair cells function to transform the sound wave into electric signals (Wang Y. et al., 2017; Liu et al., 2019b; Qi et al., 2019; Fuping Qian and Chai, 2020; Jieyu Qi, 2020; Lv et al., 2021) and are the most critical cells in the inner ear. Once damaged, hair cells have only very limited regeneration ability in mammals (Wang T. et al., 2015; Cheng et al., 2019; Tan et al., 2019; Zhang et al., 2020a; Zhang et al., 2020b; Chen et al., 2021). In the present study, we explored the effect of caffeine on hair cell damage and hearing loss. We first selected 20 and 120 mg/kg as the concentrations for two experimental groups (Mujica-Mota et al., 2014). The threshold shifts of mice treated with 20 mg/kg caffeine were not different from those of the control group (Supplementary Figure S1). However, the threshold shifts of mice in the 120-mg/kg caffeine group were higher than those of the control group. We also focused on the effect of caffeine duration on hearing. The 14-day injection caused more damage to hair cells than the 7-day injection, which indicated that hearing loss caused by caffeine was time-dependent. Western blot analyses of the cochlea verified that caffeine could induce autophagy and apoptosis in C57BL/6 mice. We also treated HEI-OC1 cells with different concentrations of caffeine (0, 0.1, 1, 5, 10, and 20 mM) for various time points (12, 24, 48, and 72 h). While treatment with 0.1 mM caffeine had no effect, treatments with caffeine concentrations greater than 1 mM caffeine significantly inhibited cell viability. In addition, 20 mM caffeine had little difference from 10 mM caffeine. Thus, we selected caffeine concentrations of 0, 1, 5, and 10 mM for 24 h for the conditions of the subsequent experiments. We cultured basilar membranes to investigate the effect of different concentrations of caffeine, and we evaluated several genes related to autophagy and apoptosis by RT-PCR and Western blot analyses. The results indicated that caffeine mainly destroyed inner hair cells. A recent study has found that coffee consumption is associated with a lower risk of disabling hearing impairment in men but not in women (Machado-Fragua et al., 2021). Moreover, previous studies have found that caffeine has definite neuroprotection in neurologic disease (Bagga and Patel, 2016; Xu et al., 2016). These differences may be due to differences in caffeine concentration and intake methods. Another review has indicated that the effect of caffeine occurs almost solely at the level of the central nervous system, suggesting that the effect of caffeine on the auditory and vestibular systems should be examined in future studies in a dose-dependent manner (Ghahraman et al., 2021). Our research revealed that the harmful effect of caffeine on cochlear hair cells was dose-dependent. Caffeine at 120 mg/kg increased auditory threshold shifts and caused hair cell loss in C57BL/6 mice, while 20 mg/kg caffeine did not cause hearing loss. Furthermore, we hypothesized that a caffeine concentration less than 1 mM may play a protective role in HEI-OC1 cells against apoptosis. It will be more difficult to find a certain protective concentration of caffeine against hearing loss, due to the large range of concentrations and diversity of hearing loss. In future studies, we will explore the possible protective effects of caffeine in the auditory system and central nervous system.

In the present research, we found that caffeine could activate the expression of SGK1 in C57BL/6 mice. SGK1 is a member of the AGC subfamily of protein kinases, a subfamily that includes proteins A, G, and C. As a ubiquitously expressed protein, SGK1 is involved in a wide variety of physiological processes and contributes to a variety of pathological conditions (Liu et al., 2017). There is a wide range of stimuli that regulate SGK expression, including dehydration, saline consumption, neuronal excitotoxicity, and DNA damage (You et al., 2004; Tang et al., 2011). In addition, SGK1 has been found to act as a switch for autophagy modulation and apoptosis in many diseases (Liu et al., 2017; Maestro et al., 2020). In our study, we utilized RNA-seq to identify different genes, and we confirmed that the expression of SGK1 increased in the cochlea after caffeine treatment both *in vitro* and *in vivo*. GSK650394 has been demonstrated *in vitro* and *in vivo* to be a specific inhibitor of SGK1, which mediates autophagy and apoptosis (Shanmugam et al., 2007; Sherk et al., 2008; Liu et al., 2017). We utilized GSK650394 and verified that caffeine induced autophagy and apoptosis by activating SGK1. Furthermore, we verified that GSK650394 protected hair cells against caffeine damage in neonatal SD rats. Thus, these findings suggested that GSK650394 might be a potential hearing protection drug, but additional experiments in different species are required in the future. According to experiments *in vitro*, we demonstrated that caffeine induced autophagy and apoptosis *via* the SGK1/HIF-1α pathway in hair cells.

HIF-1 is composed of HIF-1α and HIF-1β subunits, which are well known as oxygen-sensitive transcription factors. HIF-1α degradation is inhibited under hypoxic conditions, which facilitates the transcription of numerous genes involved in cellular adaptation to oxygen deprivation (Semenza, 2007). Importantly, a previous study has shown that the SGK1/HIF-1α signaling pathway plays a vital role in protecting renal cells from apoptosis by promoting autophagy, indicating that HIF-1α transcriptional activity is regulated by SGK1 (Xie et al., 2018). In addition, hypoxic conditions confer a great benefit to expanding cochlear stem/progenitor cells by stimulating HIF-1α (Chen et al., 2011). We verified the interaction between SGK1 and HIF-1α by co-IP. CoCl_2_ is known as a chemical-specific inducer of HIF-1α *via* simulating hypoxia (Wang M. et al., 2017; Mikami et al., 2017; Rana et al., 2019). Our results showed that GSK650394 could increase the expression of HIF-1α, while CoCl_2_ did not mediate the expression of SGK1. We speculated that HIF-1α might be a downstream signaling molecule of SGK1. Western blot data revealed that CoCl_2_ reduced the expression of Cleaved-Caspase3 and LC3B II/I after treatment with 10 mM caffeine, while CoCl_2_ increased the expression of p62 and the Bcl-2/Bax ratio. These results indicated that CoCl_2_ relieves caffeine-induced autophagy and apoptosis caused by caffeine, suggesting that HIF-1α might play an important role in mediating autophagy and apoptosis induced by caffeine.

In most normal situations, the autophagic process protects cells from apoptosis. However, cells convert to apoptosis if autophagy depletes their proteins and organelles in the case of overstimulation (Maiuri et al., 2007). As a result, apoptosis and autophagy share similar pathways at the molecular level. Many previous studies have suggested that SGK1 inhibits autophagy-dependent apoptosis (Conza et al., 2017; Liu et al., 2017; Zuleger et al., 2018). However, we found that caffeine induced autophagy and apoptosis *via* the SGK1/HIF-1α pathway in our study. These differences may be due to SGK1 playing different roles in different organisms or different diseases. Furthermore, we utilized 3-MA to explore the relationship between autophagy and apoptosis. 3-MA, which is a specific autophagy inhibitor (Wang S. et al., 2015; Shi et al., 2020; Sugawara et al., 2020), could reduce apoptosis caused by caffeine (Supplementary Figure S3). These results indicated that autophagy may lead to apoptosis when HEI-OC1 cells are treated with caffeine. In this study, we mainly confirmed the role of SGK1 in the process of autophagy and apoptosis caused by caffeine *in vitro*. For future research, we will explore the role of SGK1 in the cochlea by using knockout mice.

In summary, the present study showed that caffeine induces autophagy and apoptosis in auditory hair cells *via* the SGK1/HIF-1a pathway. Our findings provided new insights into ototoxic drugs and suggested potential therapeutic targets for the amelioration of caffeine-induced ototoxicity.

## Data Availability

The datasets presented in this study can be found in online repositories. The names of the repository/repositories and accession number(s) can be found below: NCBI [accession: SAMN22217548, SAMN22217549, SAMN22217550, SAMN22217551, SAMN22217552, SAMN22217553].
