# Supplementary material for: Caffeine Induces Autophagy and Apoptosis in Auditory Hair Cells via the SGK1/HIF-1α Pathway
Source: Front Cell Dev Biol. 2021 Nov 16;9:751012. doi: 10.3389/fcell.2021.751012 (PMC8637128; doi:10.3389/fcell.2021.751012)
Supplement: Supplementary file 1 [file DataSheet1.docx]

Supplementary Material

# Supplementary Figures and Tables

## Supplementary Figures


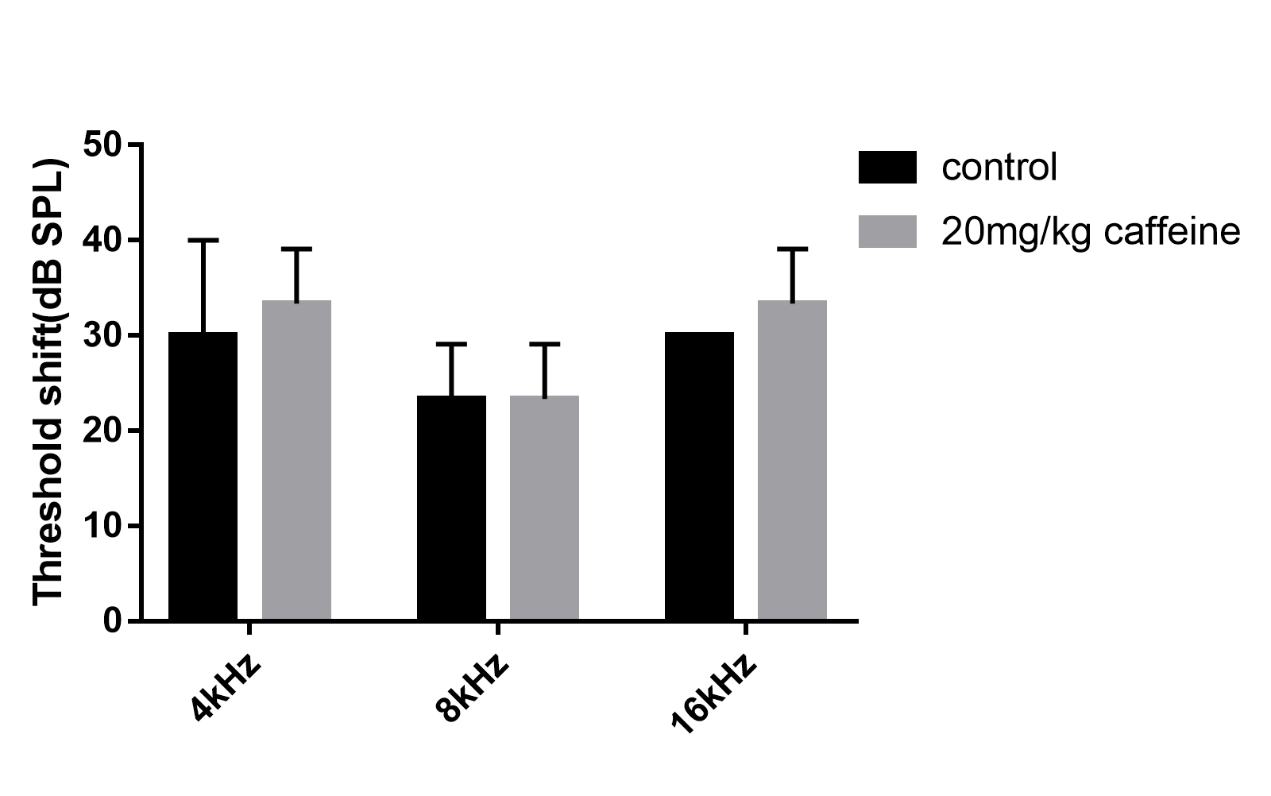


**Supplementary Figure 1.** ABR threshold shifts measured in C57BL/6 mice treated with 20 mg/kg caffeine and the control group.


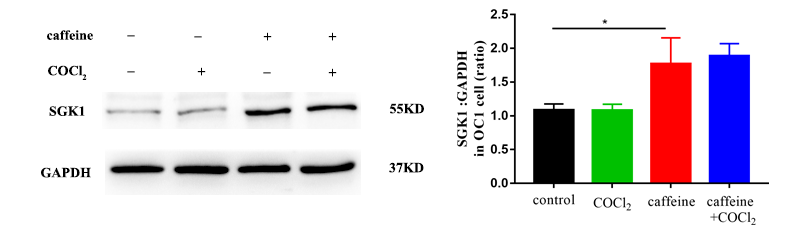


**Supplementary Figure 2.** Western blot assay was employed to investigate the expression of SGK1 in HEI-OC1 cells after 24 hours treatment with or without caffeine and CoCl_2_. GAPDH was used as the internal control. Experiments were repeated three times. Data are shown as means ± SD, **P < 0.05*.


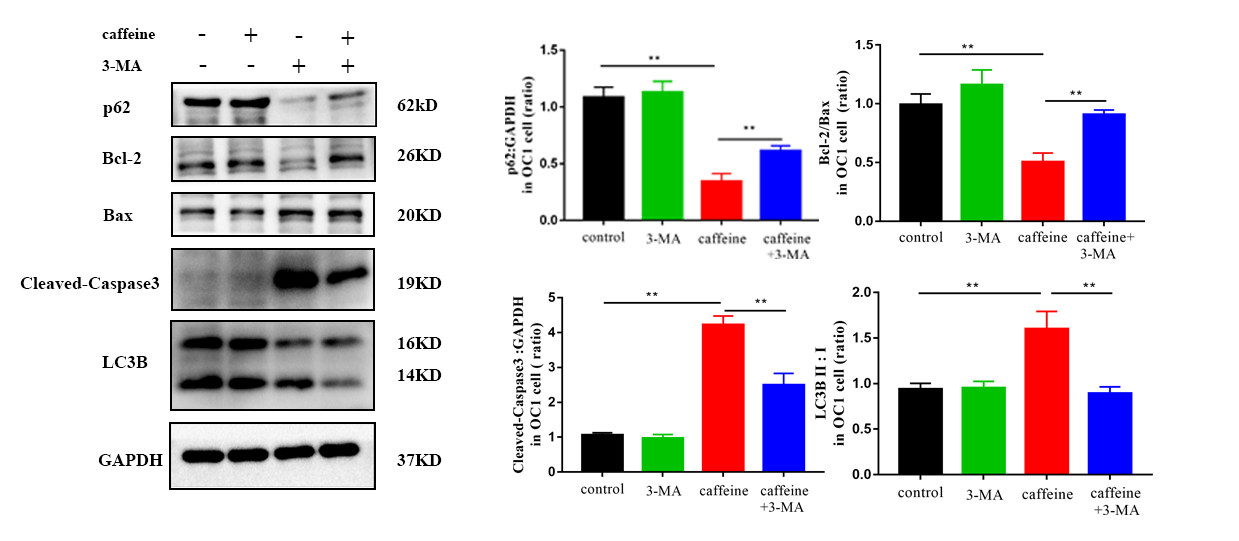


**Supplementary Figure 3.** Western blot assay was employed to investigate the expression of p62, LC3B, Bcl-2, Bax, and Cleaved-Caspase3 in HEI-OC1 cells after 24 hours treatment with or without caffeine and 3-MA. GAPDH was used as the internal control. Experiments were repeated three times. Data are shown as means ± SD, ***P < 0.01*.
